# Plasma Galectin-7 (Gal-7) and Galectin-8 (Gal-8) as Emerging Biomarkers in Psoriasis: Associations with Disease Activity and Metabolic Status

**DOI:** 10.3390/metabo16010050

**Published:** 2026-01-07

**Authors:** Julia Nowowiejska-Purpurowicz, Anna Baran, Justyna Magdalena Hermanowicz, Beata Sieklucka, Krystyna Pawlak, Dariusz Pawlak, Iwona Flisiak

**Affiliations:** 1 Department of Dermatology and Venereology, Medical University of Bialystok, Zurawia 14 St., 15-540 Bialystok, Poland; anna.baran@umb.edu.pl (A.B.); iwona.flisiak@umb.edu.pl (I.F.); 2 Department of Pharmacodynamics, Medical University of Bialystok, Mickiewicza 2C St., 15-222 Bialystok, Poland; justyna.hermanowicz@umb.edu.pl (J.M.H.); dariusz.pawlak@umb.edu.pl (D.P.); 3 Department of Monitored Pharmacotherapy, Medical University of Bialystok, Mickiewicza 2C St., 15-222 Bialystok, Poland; beata.sieklucka@umb.edu.pl (B.S.); krystyna.pawlak@umb.edu.pl (K.P.)

**Keywords:** plasma, galectin, psoriasis, galectin 7, gal-7, galectin 8, gal-8

## Abstract

**Background:** Psoriasis is a chronic, immune-mediated skin disorder characterized by accelerated epidermal turnover. Galectins are a family of carbohydrate-binding proteins that play crucial roles in various biological processes. **Methods:** This study aimed to assess the plasma concentrations of galectin 7 and 8 (gal-7 and 8) in 60 psoriatic patients compared to the control group of 30 individuals without dermatoses. **Results:** The median gal-7 plasma concentration in patients was 188.8 (11.43–1406) pg/mL, and it was significantly higher than in controls (*p* < 0.001). There was a positive correlation between gal-7 concentration and psoriasis area and severity index (PASI; R = 0.3, *p* = 0.0199), and a negative with RBC (R = −0.41, *p* < 0.001), hemoglobin concentration (R = −0.34, *p* < 0.01), total cholesterol (R = −0.38, *p* < 0.01) and LDL concentration (R = −0.36, *p* < 0.05). In contrast, gal-7 was not correlated with psoriasis duration or patients’ age or sex (*p* > 0.05). The median gal-8 plasma concentration in patients was 0.07 (0.02–0.5) ng/mL, and was significantly higher in patients than controls (*p* < 0.05). There was a positive correlation between gal-8 concentration and glucose concentration (R = 0.26, *p* < 0.05). Gal-8 concentration was not correlated with PASI, BMI, age or sex of patients (*p* > 0.05). We also analyzed the receiver operating characteristic (ROC) curve to evaluate the predictive power of gal-7 and 8 for psoriasis. Gal-7 achieved statistical significance in predicting psoriasis and had an area under the curve (AUC) value of 0.842 (*p* < 0.001), a sensitivity of 80%, and a specificity of 86.7%, whereas gal-8 had an AUC value of 0.644 (*p* = 0.025), a sensitivity of 81%, and a specificity of 47%. **Conclusions:** Gal-7 and gal-8 could potentially serve as psoriasis biomarkers, whereby gal-7 could also serve as a marker of its severity. Future studies are needed to clarify their actual role or potential as therapeutic targets in psoriasis. Understanding their precise functions may open new perspectives for personalized treatment strategies in psoriatic patients.

## 1. Introduction

**Figure 1:**
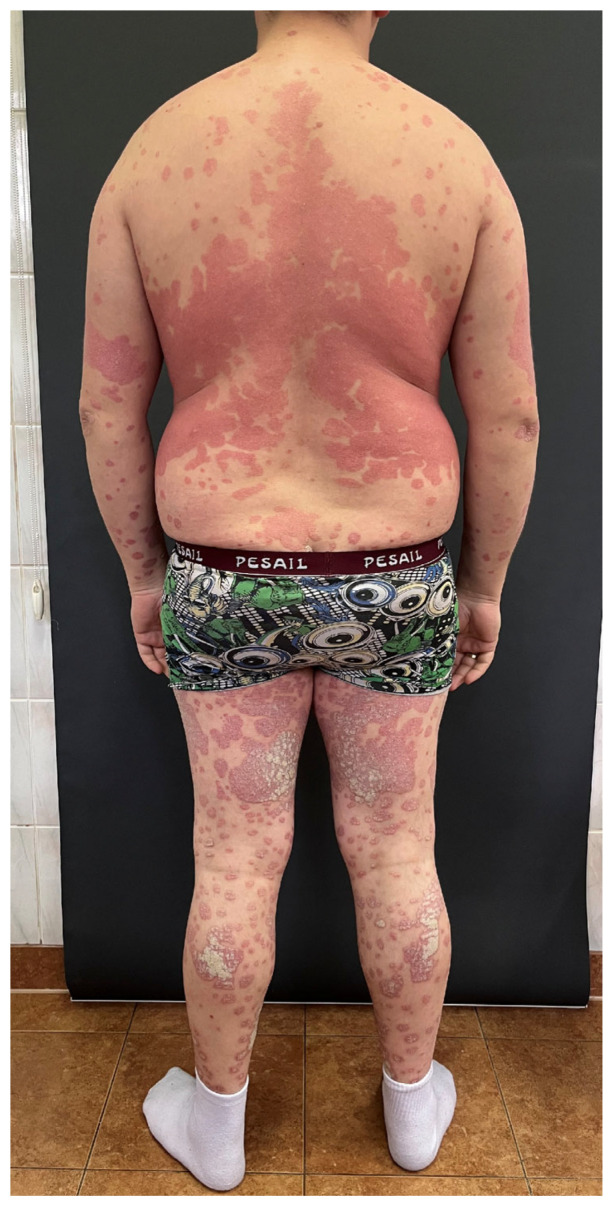
A patient with extensive lesions of a plaque psoriasis.

Psoriasis is a chronic, immune-mediated skin disorder with genetic susceptibility, characterized by accelerated epidermal turnover, leading to improper keratinocyte proliferation and differentiation, resulting in the development of psoriatic plaques on the skin [1]. Genome-wide linkage studies of families suffering from psoriasis have identified at least 60 chromosomal loci linked to psoriatic susceptibility, with PSORS1 being the most prominent locus [1]. The condition is driven by an overactive immune response, particularly involving Th cells and inflammatory cytokines like TNF-α and interleukins (particularly IL-17 and IL-23), which trigger excessive keratinocyte proliferation [1,2]. The influence of environmental factors is also significant, as they may trigger psoriasis for the first time or exacerbate the already existing lesions. These include particularly trauma, infections, certain medications, or alcohol [1]. Although psoriasis prevalence may differ across geographical regions, the average reported frequency worldwide is estimated at 2% [1]. It may appear at any age, and the frequency seems to be equal in both sexes [2]. Psoriasis has several forms, with plaque psoriasis being the most common [2]. It commonly affects areas such as the extensor surfaces of the elbows and knees, scalp, and lower back, but can occur anywhere on the body, including the nail unit (Figure 1).

The diagnosis is made based on the clinical presentation, supported by dermoscopy and, in uncertain cases, confirmed by pathology [2]. Psoriasis is incurable; however, nowadays there are numerous efficacious therapeutic options. The treatment depends on the severity and localization of skin lesions. Available options include topical agents (particularly betamethasone + calcipotriol, topical steroids, topical calcineurin inhibitors), phototherapy (narrow band UVB, psoralen UVA), classic systemic agents (methotrexate, cyclosporin A, acitretin, apremilast, dimethyl fumarate), and biological treatment (TNFα, IL-17, IL-23 inhibitors) [1]. Psoriasis is also associated with a broad comorbidity, of which psoriatic arthritis and metabolic syndrome have been most proven [2]. Notably, psoriasis generates a high economic burden; in 2021, it was reported to be 148.85 billion dollars, composed of 95.15 billion dollars for healthcare costs and 53.70 billion dollars in productivity losses [3]. This illustrates how important this problem is and how necessary it is to deepen our knowledge about its monitoring and treatment.

Galectins, also called S-type lectins in the past, are a family of carbohydrate-binding proteins characterized by their affinity for β-galactoside carbohydrates and a conserved carbohydrate recognition domain (CRD) [4,5]. Several types of galectins have been discovered; however, they may vary in occurrence between species. In humans, the following galectins have been identified to date: 1, 2, 3, 4, 7, 8, 9, 10, 12, 13, 14, 16 [4]. Interestingly, despite their diversity, galectins retain a certain evolutionary primary structure, as well as gene and CRD structure [5]. They play crucial roles in various biological processes, including cell adhesion, immune response modulation, apoptosis, and inflammation; however, their properties are dependent not only on their particular structure but also on the molecules the galectins bind with [6]. Galectins are synthesized in the cytoplasm [5] and expressed in a wide range of tissues and are often secreted into the extracellular space despite lacking classical secretion signals [7]. Their ability to crosslink glycoproteins on cell surfaces allows them to influence signaling pathways and cellular communication. Dysregulation of galectin expression has been linked to numerous diseases, including cancer, infections, fibrosis, and autoimmune disorders [7], making them important targets for therapeutic research.

Galectin 7 (gal-7) is a prototypical galectin, which is predominantly expressed in stratified epithelia, hair follicles, and thymic Hassall’s corpuscles [5,8]. This galectin has been initially reported to participate in the healing of skin injury, either mechanical or after exposure to ultraviolet B radiation [8]. It has even been suggested as a marker of skin barrier impairment [9]. Moreover, gal-7 involvement in the regulation of keratinocyte proliferation and differentiation has been reported (through the JNK1-miR-203-p63 pathway) [8], along with its potential role as a mediator of cutaneous carcinogenesis [10].

Galectin 8 (gal-8) is a tandem-repeat-type galectin that is characterized by two different carbohydrate-recognition domains combined by a linker peptide. Gal-8 does not possess the N-terminal signal sequence; hence, it is secreted in an atypical secretion mechanism. It is expressed in the lungs, spleen, liver, kidneys, and cardiac muscles [7]. As for the properties, gal-8 influences innate and adaptive paths of immune response [11]. It has been implicated in the antibacterial and antiviral defense, and as a protective agent in neurodegenerative processes; however, on the other hand, it plays a promoting role in the regulation of malignant tumors’ growth and metastases [7].

Psoriasis is related to immunological disturbances, both in innate and adaptive immunity [4]. The hallmark of psoriasis is increased keratinocyte proliferation and improper differentiation. The epidermal turnover time, which normally lasts about 28 days, is shortened in psoriasis down to even 4 days [12]. Considering the role of gal-7 in the proliferation and differentiation of keratinocytes, and gal-8—in the immune response, we decided to study these proteins in psoriatic patients.

## 2. Materials and Methods

### 2.1. Participants

A total of 60 Caucasian patients (21 women and 39 men) with active plaque psoriasis were recruited for this study, with a mean age of 49 ± 2.3 years. These individuals were compared to a control group of 30 volunteers (20 men and 10 women) matched for age (mean age 47 ± 2.5), race, sex, and BMI, who came from the same geographical area and had no skin disorders and no family history of psoriasis. All participants provided written informed consent before joining the study. The inclusion criteria were: plaque psoriasis diagnosis made by a dermatologist, age over 18, and no systemic antipsoriatic treatment. Individuals were excluded if they were under 18 years old, pregnant, had forms of psoriasis other than plaque type, followed special diets, had taken oral medications within the previous 3 months, or had infectious diseases, autoimmune conditions (other than psoriasis), kidney disease, or cancer. Psoriasis severity was evaluated using the Psoriasis Area and Severity Index (PASI) by the same dermatologist for consistency. PASI includes the assessment of erythema, infiltration, and scaling, as well as skin lesions area, in four anatomic areas (head, trunk, upper and lower extremities). The higher the score, the more severe the lesions (the minimal score is 0, the maximal is 72). Based on PASI scores, patients were categorized into three groups: mild (PASI < 10), moderate (PASI 10–20), and severe (PASI > 20). Patients were also divided according to disease duration: less than 15 years or more than 15 years. Body mass index (BMI) was calculated using the standard formula (weight/height^2^), and standard numerical ranges were used for its interpretation. Before the study began, laboratory tests were performed to assess C-reactive protein (CRP), complete blood count, fasting glucose, lipid profile, and liver and kidney function. The study was approved by the Bioethics Committee of the Medical University of Bialystok, Poland (Approval No. APK.002.19.2020) and conducted in accordance with the Declaration of Helsinki.

### 2.2. Plasma Collection and Experiment

Fasting blood samples were collected into vacuum tubes and centrifuged at 2000× *g* for 10 min. The resulting plasma was stored in a freezer at –80 °C until analysis. Routine laboratory methods were used to measure the basic parameters (complete blood count, CRP, glucose, liver enzymes, lipid profile, and kidney function parameters). Plasma levels of gal-7 and gal-8 were determined using enzyme-linked immunosorbent assay (ELISA) kits from Cloud Clone^®^ (Houston, TX, USA; SEA307Hu, SEA308Hu).

The measurement principle was based on a sandwich reaction. In the first stage, 100 µL of standards and samples were added to each well of a plate coated with an antibody specific for gal-7 or gal-8, respectively. Subsequently, the plate was incubated for 1 h at 37 °C. Then 100 µL of Detection Reagent A was added to the plate and incubated for 60 min at 37 °C. In the next step, the plate was rinsed three times, and 100 µL of Detection Reagent B was added to each well, which was then incubated for 30 min at 37 °C. The plate-washing steps were performed using an Elx-50 automated microplate washer (BioTEK^®^, Agilent, Santa Clara, CA, USA). After the incubation period, the plate was washed five times. In the next step, 90 µL of Substrate was added to each well, and incubated for 20 min at 37 °C. The enzymatic reaction was terminated by adding 50 µL of STOP Solution (sulphuric acid solution). The activity of gal-7 and gal-8 was measured photometrically at 450 nm using a Multiscan FC microplate reader (ThermoScientific^®^, Waltham, MA, USA). All analyses were performed by the same technician under standardized conditions.

### 2.3. Statistical Evaluation

The distribution of data was evaluated using the Shapiro–Wilk test. Normally distributed variables were analyzed using the Student’s *t*-test or one-way ANOVA and presented as mean ± standard deviation. Non-normally distributed data were shown as median (full range) and analyzed with the Mann–Whitney or Kruskal–Wallis tests. Correlations between variables were assessed using Spearman’s rank correlation. All statistical analyses were performed using GraphPad Prism 9.4, and results were considered statistically significant at *p* < 0.05.

**Figure 2:**
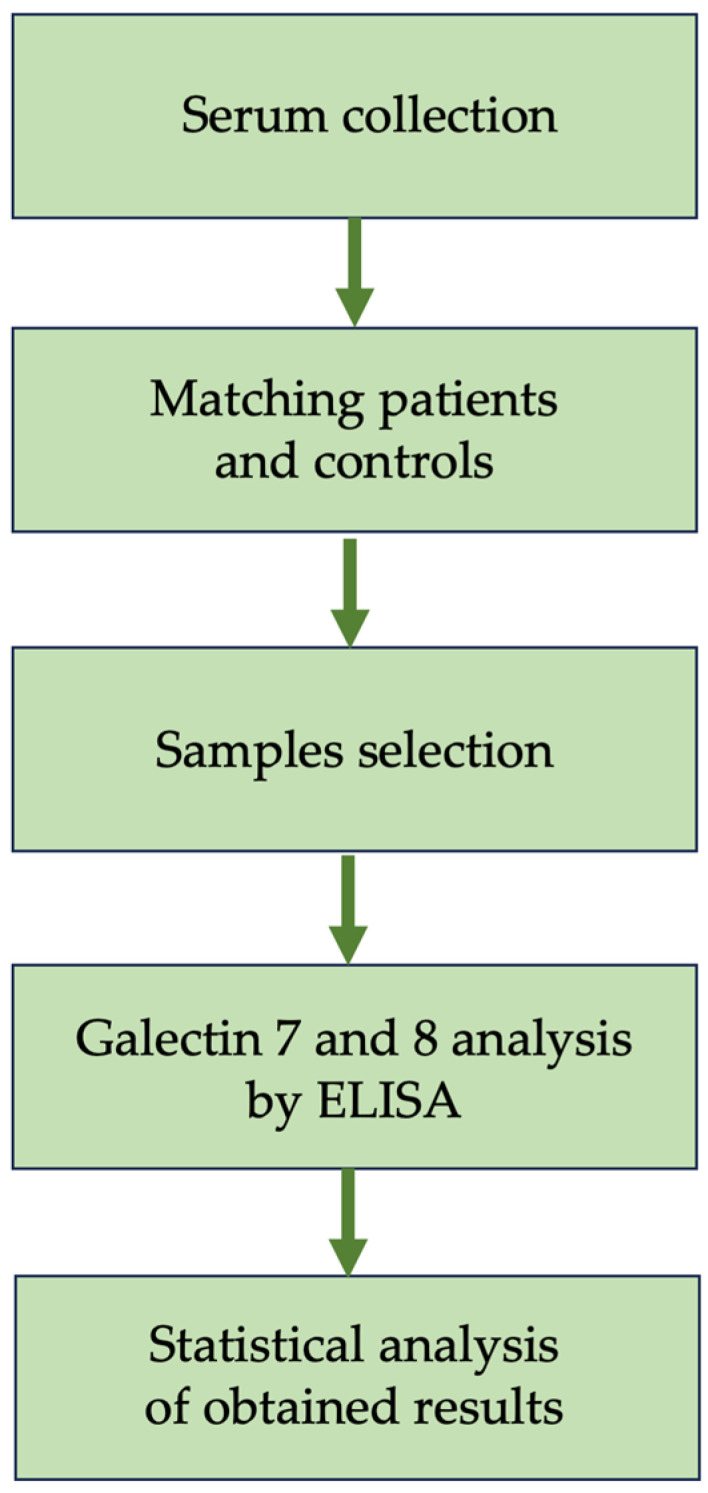
The flow diagram of the conducted study.

Figure 2 presents the flow diagram of the conducted study (Figure 2).

## 3. Results

A total of 60 patients with psoriasis and 30 volunteers without dermatoses were included in the analysis. There was no statistically significant difference in age or sex between the groups (*p* > 0.05). The mean BMI of patients was 27.6 ± 0.8, which indicates overweight. It was not significantly different from the mean BMI of individuals from the control group, 25.7 ± 0.77 (*p* > 0.05), which also indicates overweight (Table 1). The mean psoriasis duration in patients was 18.7 years.

**Table 1:** The basic characteristics of the participants.

Parameter	Control Group (30)	Study Group (60)	*p*-Value
Sex (M/F)	20/10	39/21	>0.05
Age [years]	47 ± 2.5	49 ± 2.3	>0.05
BMI	25.7 ± 0.77	27.6 ± 0.8	>0.05

Data presented as mean ± SD; BMI, body mass index; M, males; F, females.

### 3.1. Galectin 7

**Figure 3:**
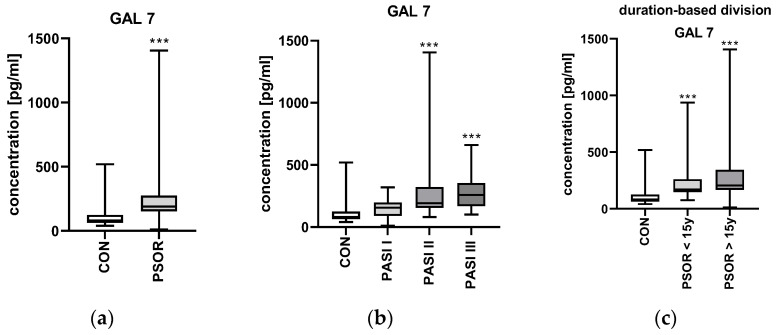
Galectin 7 plasma concentration in psoriatic patients and controls: (**a**) absolute concentration; (**b**) division based on psoriasis severity; (**c**) division based on the duration of psoriasis. *** means a statistically significant difference compared to controls with *p* < 0.001.

The median gal-7 plasma concentration in patients was 188.8 (11.43–1406) pg/mL. It was significantly higher in patients than in controls—82.34 (41.15–519.7) pg/mL (*p* < 0.001) (Figure 3a).

After the division of patients based on the psoriasis severity expressed by PASI, there was no significant difference in gal-7 plasma concentration between the particular PASI subgroups (*p* > 0.05), although the highest median values were found in the group with the most severe lesions (Figure 3b).

After the division of patients according to the duration of psoriasis, there was no statistically significant difference between the subjects with long-lasting psoriasis (more than 15 years) and subjects with shorter disease duration (less than 15 years); (*p* > 0.05) (Figure 3c).

**Figure 4:**
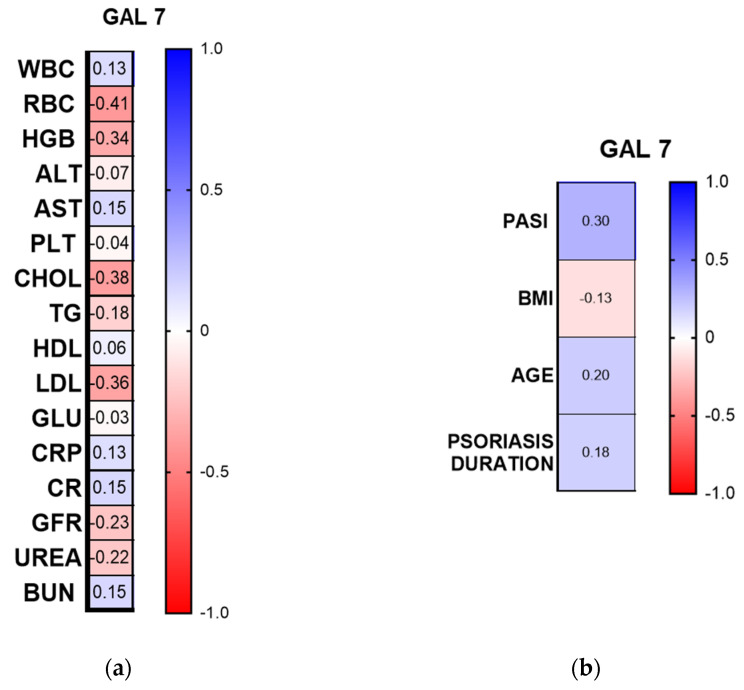
Spearman’s rank correlation heat-map. Numbers represent Spearman’s rank value. ALT, alanine aminotransferase; AST, aspartate aminotransferase; BUN, blood urea nitrogen; CR, creatinine; GLU, fasting glucose; GFR, glomerular filtration rate; CHOL, total cholesterol; HDL, high-density lipoprotein; LDL, low-density lipoprotein; RBC, red blood cells; WBC, white blood cells; PLT, platelets; HGB, hemoglobin; TGs, triglycerides. (**a**) correlations with laboratory parameters; (**b**) correlations with clinical and demographic parameters.

Among the laboratory parameters, there was a negative correlation between gal-7 concentration and RBC (R = −0.41, *p* < 0.001, CI 95%), HGB concentration (R = −0.34, *p* < 0.01, CI 95%), total cholesterol (R = −0.38, *p* < 0.01, CI 95%), and LDL concentration (R = −0.36, *p* < 0.05, CI 95%) (Figure 4a).

Gal-7 concentration was positively correlated with PASI (R = 0.3, *p* = 0.0199, CI 95%), but there was no significant correlation with psoriasis duration, patients’ age, or BMI (*p* > 0.05) (Figure 4b).

**Figure 5:**
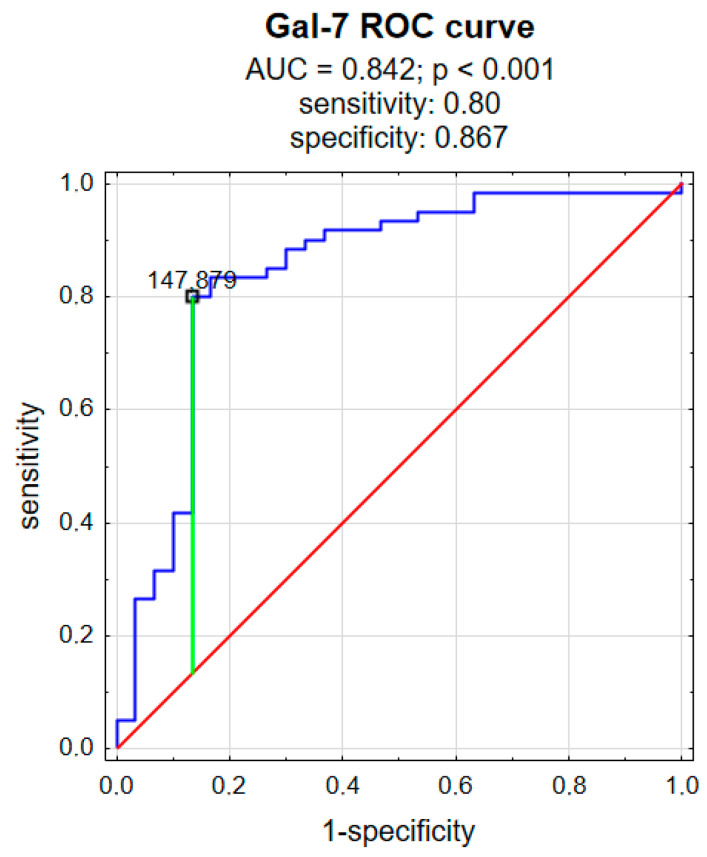
ROC curve for galectin 7. red curve means the random classifier, blue curve means the actual test, green curve means the chosen cut-off.

We also analyzed the receiver operating characteristic (ROC) curve to evaluate the predictive power of gal-7 for psoriasis. Gal-7 achieved statistical significance in predicting psoriasis and had an area under the curve (AUC) value of 0.842 (*p* < 0.001), a sensitivity of 80%, and a specificity of 86.7% (Figure 5).

### 3.2. Galectin 8

**Figure 6:**
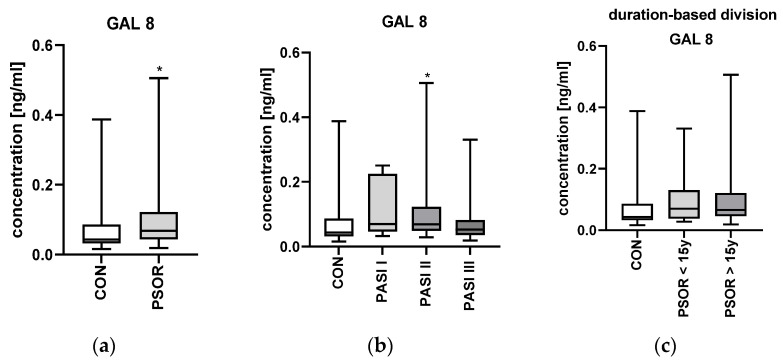
Galectin 8 plasma concentration in psoriatic patients and controls: (**a**) absolute concentration; (**b**) division based on psoriasis severity; (**c**) division based on the duration of psoriasis. * means a statistically significant difference compared to controls with *p* < 0.05.

The median gal-8 plasma concentration was 0.07 (0.02–0.5) ng/mL. It was significantly higher in patients than in controls—0.04 (0.02–0.39) ng/mL (*p* < 0.05) (Figure 6a).

After the division of patients based on the psoriasis severity expressed by PASI, there was no significant difference in gal-8 concentration between the particular subgroups (*p* > 0.05) (Figure 6b).

After the division of patients according to the duration of psoriasis, there were no significant differences between patients with short and long-lasting psoriasis regarding gal-8 concentrations (*p* > 0.05) (Figure 6c).

**Figure 7:**
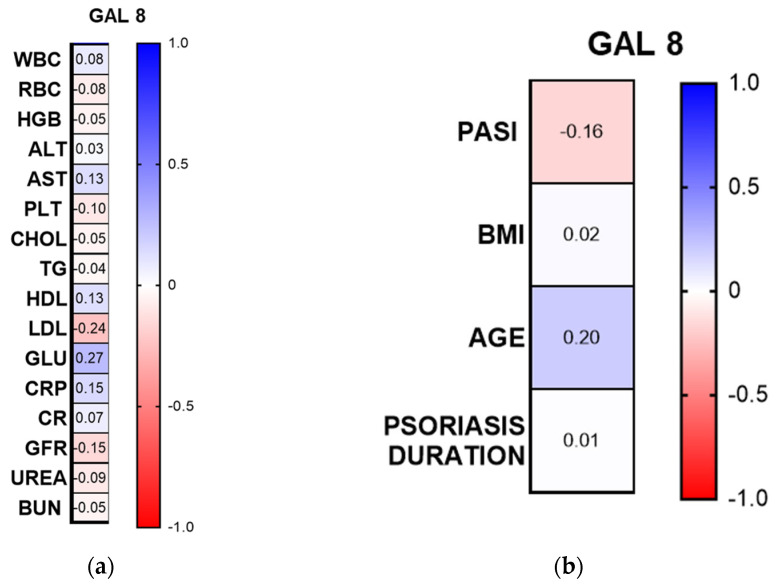
Spearman’s rank correlation heat-map. ALT, alanine aminotransferase; AST, aspartate aminotransferase; BUN, blood urea nitrogen; CR, creatinine; GLU, fasting glucose; GFR, glomerular filtration rate; CHOL, total cholesterol; HDL, high-density lipoprotein; LDL, low-density lipoprotein; RBC, red blood cells; WBC, white blood cells; PLT, platelets; HGB, hemoglobin; TGs, triglycerides. (**a**) correlations with laboratory parameters; (**b**) correlations with clinical and demographic parameters.

Among the laboratory parameters, there was a positive correlation between gal-8 concentration and glucose concentration (R = 0.27, *p* < 0.05, CI 95%) (Figure 7a).

Gal-8 concentration was not correlated with psoriasis duration, psoriasis severity in PASI, or BMI and age of patients (*p* > 0.05) (Figure 7b).

**Figure 8:**
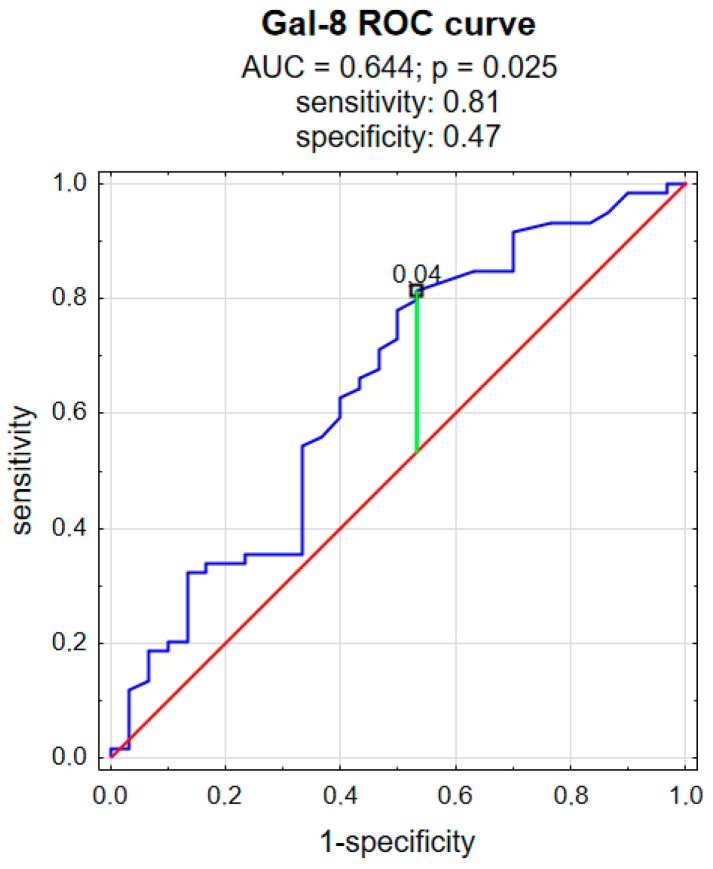
ROC curve for galectin 8. red cuve means the random classifier; blue curve means the actual test; green curve means the chosen cut-off point.

ROC analysis was performed to evaluate the predictive power of gal-8 for psoriasis. As shown in Figure 8, gal-8 achieved statistical significance in predicting psoriasis and had an AUC value of 0.644 (*p* = 0.025), a sensitivity of 81%, and a specificity of 47% (Figure 8).

## 4. Discussion

So far, almost all of the twelve galectins described in humans [4] have been studied in psoriasis, except for gal-13, gal-14, and gal-16 [13,14,15,16,17]. Recent studies suggest that galectins act as important regulators of the inflammatory microenvironment and may influence both the cutaneous and systemic manifestations of psoriasis. Our team analyzed the role of gal-1, 2, 3, 4, 10, 12 in the past [15,16,17]. All of them were significantly elevated in psoriatic patients’ sera compared to subjects without dermatoses. Gal-1, 2, 3, 12 have been suggested as the markers of cardiometabolic complications in psoriatic patients [15,16]. Hereby, we present the outcomes regarding gal-7 and gal-8 in psoriasis, which seem to exert an opposite influence on its pathogenesis. Understanding the balance between these galectins may provide new insights into the molecular mechanisms of psoriasis and potential therapeutic targets.

Gal-7 is present both in the nucleus and cytoplasm of epidermal cells [8], as well as in the extracellular space [18]. Previous studies indicated that gal-7 probably inhibits keratinocyte proliferation because gal-7-deficient mice presented an increased expression of Ki-67 on epidermal cells [8,19]. Its role in skin diseases had already been demonstrated. It is involved in the pathogenesis of atopic dermatitis, non-melanoma skin cancers, or Stevens-Johnson syndrome/toxic epidermal necrolysis [9,10,20]. Gal-7 had already been also analyzed in psoriatic patients; however, the study concerned the mRNA or the expression of this galectin in the skin samples [18], not circulating gal-7 concentrations. Gal-7 mRNA has been reported to be downregulated in psoriatic skin [21]. Chen et al. found decreased expression of gal-7 in the psoriatic plaque compared to the non-lesional patients’ skin and compared to the healthy skin of subjects without psoriasis; similar observations were made on the mouse model of psoriasis [18]. The same scientists further induced a psoriasis-like skin inflammation by the injections of IL-23 to the skin and observed decreased expression of gal-7 in the inflamed area, and assessed the cytokine interplay. IL-17A and TNF-α decreased the gal-7 expression in the spontaneously immortalized human keratinocyte cell lines (HaCaT) and human epidermal keratinocytes, neonatal (HEKn) cell lines [18]. They concluded that the downregulation of gal-7 in psoriatic epidermis leads to epidermal hyperplasia and skin inflammation [18].

Although earlier studies demonstrated decreased gal-7 expression in psoriatic epidermis, we found significantly elevated plasma gal-7 concentrations in psoriatic patients compared to subjects without skin diseases (*p* < 0.001). What is more, gal-7 appeared to be positively correlated with PASI, which means that patients with higher severity of psoriasis had higher gal-7 concentration, so it could be further investigated as a psoriasis severity laboratory marker. The ROC curve for gal-7 demonstrates good discriminative performance between the two groups (patients vs. controls). The curve lies well above the diagonal reference line, indicating performance substantially better than chance.

This apparent discrepancy between the previous studies and ours may be explained by several hypotheses. Firstly, we investigated the systemic concentration of gal-7, not a local mRNA expression, which reflects a compartmental production. Considering psoriasis is a systemic inflammatory disease, elevated plasma gal-7 could be a systemic response rather than a direct reflection of cutaneous expression. Another aspect that could be taken into account is that gal-7 may also originate from other epithelial tissues or immune cells, which respond differently to systemic cytokines. Systemic inflammation may perhaps promote gal-7 release elsewhere, increasing circulating levels while skin-specific pathways suppress local transcription. Considering all of the above, the matter of gal-7 in psoriasis requires further investigation. The role of plasma gal-7 is of special interest, since its collection is feasible, relatively non-invasive, and possible in daily clinical practice, which makes it a potential biological material for testing.

In our study, gal-7 plasma concentration was negatively correlated with the number of red blood cells (RBCs) and hemoglobin concentration. As mentioned above, psoriasis is a condition associated with systemic inflammation, which may undoubtedly influence the erythrocytes and hemoglobin, as it happens in many chronic diseases [22]. Chronic stress, such as a chronic disease, including psoriasis, can lead to the increased synthesis of reactive oxygen species (ROS), resulting in oxidative stress. ROS can exert a negative influence on both RBC and hemoglobin. RBC are highly prone to oxidative damage because of their role in oxygen transport and their high content of oxidation-sensitive biomolecules. ROS can cause lipid peroxidation and compromise membrane integrity, as well as alter hemoglobin’s structure and function. Dysfunctional RBC have poorer performance and survival, which can lead to anemia [23]. The exact incidence of anemia in patients with psoriasis is unknown, and there is a paucity of data on this matter; however, as psoriasis is now considered a chronic systemic inflammatory disease, anemia may occur more frequently in such individuals than in the general population. Currently, there is no clear evidence on how gal-7 could be engaged in anemia pathophysiology, hence more studies are required to investigate this issue. Nevertheless, there are observations on elevated gal-7 plasma concentrations in patients with multiple myeloma, and they are correlated negatively with hemoglobin and hematocrit [24]. Moreover, we noted that elevated gal-7 was also associated with more severe skin lesions. Therefore, the results are consistent in that patients with psoriasis, especially severe, may have lower red blood cell parameters.

An unexpected observation in our investigation is that gal-7 correlated negatively with total cholesterol and LDL concentration. It seems surprising considering that psoriasis is known to be associated with cardiometabolic complications [2], and in our previous studies, we demonstrated the role of several other galectins in these comorbidities [15]. However, in chronic inflammatory diseases (which undoubtedly include psoriasis), a severe systemic inflammatory condition may lower circulating total cholesterol and LDL. Studies have shown that LDL concentrations are often decreased, whereas VLDL increased, because there is an exchange of triglycerides between triglyceride-rich lipoproteins and LDL [25]. The potential explanation is that pro-inflammatory cytokines, such as TNF-α, IL-1β, and IL-6 (which are also increased in psoriatic patients), can increase hepatic LDL receptor activity and enhance cholesterol uptake; IL-1 also suppresses cholesterol synthesis and decreases cholesterol and apolipoprotein B secretion [26].

On the other hand, gal-7 seems not to be associated with psoriasis duration or patients’ age, sex, and BMI.

Gal-8 has also already been studied in psoriasis, but, like gal-7, only in tissue samples. In the past, it has been demonstrated that gal-8 mRNA levels were significantly higher in psoriatic plaques than in unlesional skin [21], and that gal-8 expression was more intense in the psoriatic epidermis [27]. On the mouse model, where psoriasis-like inflammation was induced by the injections of IL-23, gal-8 mRNA was also induced [27]. The authors concluded that gal-8 must be involved in the stimulation of keratinocyte proliferation in psoriasis, in which IL-17 mediates [27]. In contrast to gal-7, our findings concerning gal-8 are consistent with what had been previously observed—patients with psoriasis had significantly higher gal-8 plasma concentrations. Taking all this into account, both the phenomena at the molecular level and laboratory studies, it can be concluded that gal-8 may actually be a marker of psoriasis. The ROC curve for gal-8 also demonstrates good discriminative performance between the two groups (patients vs. controls). However, unlike gal-7, gal-8 does not seem to be associated with psoriasis severity expressed by PASI, so it cannot serve as a marker of disease activity.

Gal-8 was positively associated with glucose concentration, which could indicate that it is involved in the promotion of carbohydrate metabolism disturbances in this group of patients, potentially due to metabolic dysregulation or cytokine stress that disrupts glucose homeostasis. Psoriasis was linked to metabolic syndrome many years ago [28], and patients with psoriasis are known to suffer from diabetes mellitus more often. According to the meta-analysis by Mamizadeh et al. the risk of type 2. diabetes mellitus in psoriatics is 69% [29]. There are several genes identified that are associated with both psoriasis and diabetes. Moreover, as has been mentioned many times, psoriasis is associated with systemic inflammation, improper adipokine concentrations, and dysregulation of antioxidant mechanisms, which altogether increase the risk of diabetes mellitus in such individuals [30]. However, to the best of our knowledge, there is no study on the role of gal-8 in diabetes mellitus, insulin resistance, or dyslipidemia. Most research on gal-8 focuses on cellular functions and immune response, rather than carbohydrate or lipid metabolism, so further studies are needed.

As for demographic and clinical parameters, gal-8 does not seem to be associated with psoriasis duration or patients’ age, sex, and BMI, similar to gal-7, suggesting that circulating levels of this galectin are independent of general patient characteristics, body mass, and disease chronicity.

Despite thorough research, we were unable to find any information on the role of gal-8 in skin diseases other than psoriasis.

While discussing the potential use of galectins, we must also take into account other potential explanations for their increased concentration. One of them could be the tissue-to-plasma shift or damage to the particular cells. As psoriasis is associated with a chronic inflammatory condition, it may lead to the increased release of galectins from other epithelia or different organs, resulting in their increased plasma concentration. The activation of particular cytokine pathways, as mentioned earlier, may also influence galectins.

A limitation of this study is that it has a relatively low number of participants, who are of only one ethnicity. Moreover, it is a cross-sectional study at only one time point, and no follow-up of galectin concentration over time or after the therapy. The study involved only one source of galectin—plasma. Another limitation could be the assessment of the glucose concentration only, in the absence of other carbohydrate metabolism parameters, such as insulin, HOMA-IR, HbA1c, or adipokines. Outcomes could also be influenced by patients’ metabolic comorbidities, which are, however, difficult to exclude considering the association between psoriasis and metabolic syndrome.

## 5. Conclusions

This study examined the potential role of galectins 7 and 8 in psoriasis by measuring their plasma concentrations. It was demonstrated that psoriatic patients have significantly higher galectin 7 and 8 plasma concentrations compared to persons without dermatoses. The ROC curves for galectin 7 and 8 also demonstrate good discriminative performance between the patients and controls. Galectin 7 was positively associated with psoriasis severity expressed by PASI, which may suggest its use as a psoriasis activity marker, whereas galectin 8 was not. Galectin 7 was negatively associated with red blood cell count and hemoglobin concentration, as well as total cholesterol and LDL concentration, which may reflect the influence of chronic systemic inflammation on the erythrocytes and lipid metabolism. Galectin 8 was positively associated with glucose concentration, which could indicate that it is involved in the promotion of carbohydrate metabolism disturbances; however, this matter requires further research. Neither galectin 7 nor galectin 8 was associated with psoriasis duration, age, sex, or BMI of patients. Future studies are needed to clarify whether galectins undoubtedly serve primarily as mediators, biomarkers, or potential therapeutic targets in psoriasis. Understanding their precise functions may open new perspectives for personalized treatment strategies in psoriatic patients.

## Data Availability

Data available upon the request from the corresponding author.

## Abbreviations

The following abbreviations are used in this manuscript:

ALT	alanine aminotransferase
AST	aspartate aminotransferase
BUN	blood urea nitrogen
CRD	carbohydrate recognition domain
CRP	C-reactive protein
gal	galectin
GLU	glucose
GFR	glomerular filtration rate
HGB	hemoglobin
IL	interleukin
HDL	high-density lipoprotein
LDL	low-density lipoprotein
PASI	psoriasis AREA and severity index
PLT	platelets
RBC	red blood cells
TNF	tumor necrosis factor
WBC	white blood cells
